# A new tool to determine the cellular metabolic landscape: nanotechnology to the study of Friedreich’s ataxia

**DOI:** 10.1038/s41598-019-55799-z

**Published:** 2019-12-17

**Authors:** Tommaso Vannocci, Simone Dinarelli, Marco Girasole, Annalisa Pastore, Giovanni Longo

**Affiliations:** 1 UK Dementia Research Institute at King’s College London, London, SE5 9RT United Kingdom; 2 The Wohl Institute at King’s College London, London, SE5 9RT United Kingdom; 3 Istituto di Struttura della Materia – CNR, Via del Fosso del Cavaliere 100, 00133 Rome, Italy

**Keywords:** Nanobiotechnology, Neurological disorders, Neurological disorders, Diseases, Sensors

## Abstract

Understanding the cell response to oxidative stress in disease is an important but difficult task. Here, we demonstrate the feasibility of using a nanomotion sensor to study the cellular metabolic landscape. This nanosensor permits the non-invasive real-time detection at the single-cell level and offers high sensitivity and time resolution. We optimised the technique to study the effects of frataxin overexpression in a cellular model of Friedreich’s ataxia, a neurodegenerative disease caused by partial silencing of the *FXN* gene. Previous studies had demonstrated that *FXN* overexpression are as toxic as silencing, thus indicating the importance of a tight regulation of the frataxin levels. We probed the effects of frataxin overexpression in the presence of oxidative stress insults and measured the metabolic response by the nanosensor. We show that the nanosensor provides new detailed information on the metabolic state of the cell as a function of time, that agrees with and complements data obtained by more traditional techniques. We propose that the nanosensor can be used in the future as a new and powerful tool to study directly how drugs modulate the effects of oxidative stress on Friedreich’s ataxia patients and, more in general, on other neurodegenerative processes.

## Introduction

Understanding the primary causes of a disease is central to design ways to prevent pathology. This is not a problem in infective diseases in which the primary causative agent, when known, is clear and determinant. More difficult is to deal with neurodegenerative diseases of which we have still only a partial and confusing understanding. We for instance ignore when the different events that are often associated with neurodegeneration such as inflammation, oxidative stress, metabolic changes, occur. Currently, the only way to follow these processes is to take reads of different biomarkers from patients or animal model samples at specific quantised time points and use them as objectively measurable parameters that reflect the pathogenic processes or the response to therapeutic pharmacologic intervention. This approach is appropriate in many cases but can miss completely the early events of disease development or confuse primary causes with secondary effects^1^. Conventional bulk biological assays are also limited by the intrinsic cellular heterogeneity in gene, protein and metabolite expression and investigate the cellular status only indirectly. In search for new methodologies able to assist both basic studies of molecular mechanisms, drug discovery and diagnosis, we implemented the use of a novel bio-nanosensor, named nanomotion sensor, a device able to perform real-time, single-cell correlated measurements of different cellular nanoscale biomotions^2^. This innovative methodology combines conventional bio-investigation techniques and nanomechanical oscillators, typically an AFM cantilever^3^, to obtain with modest costs, a small and manageable device that can transduce in measurable fluctuations the nanoscale metabolically-related cellular movements or vibrations^4^. These signals can be used to study the cellular response to physical or chemical external stimuli. The response reflects in turns the metabolic activities of the cell and gives us a quantitative measure of the viability of the cellular specimen^5^. The nanosensor can be used to study cellular reactions and cytotoxicity an order of magnitude faster and using 100-fold smaller volume of reagents when compared to conventional analyses: this methodology allows monitoring the response to external stimuli in the order of hours, i.e. way before what can be done by the conventional high-throughput bulk assays, and provides a platform of unparalleled sensitivity and time resolution to monitor  *in continuum* different biological systems, such as bacteria, yeast and mammalian cells^6,7^. The approach has already been used to investigate a large variety of molecular^8^, cellular^9^, microbiological^10,11^ and clinical problems^12^. We have for instance used the nanosensor to study single neuroblastoma cells exposed to extracellular monomeric and amyloid *α*-synuclein, the protein involved in Parkinson disease^9,13^. By combining the nanosensor with fluorescence microscopy, we demonstrated that *α*-synuclein aggregates lead to cooperative cytotoxic effects and aggregate-induced loss of cellular membrane integrity. We also employed the nanomotion sensor to characterize the status of Calmette-Guérin *Mycobacterium* bacilli and non-tuberculous *Mycobacteria* treated with different antibiotics, to study the effects and efficacy of the drugs^11^. We convincingly showed how the high-speed and high-sensitivity of the nanosensor provide a rapid and reliable method to study the response of the bacillus to drugs.

Here, we implemented the nanosensor to study human cells modified to switch on/off the FXN gene implicated in Friedreich’s ataxia (FRDA)^14^. This is an autosomal recessive disease caused by trinucleotide repeats expansion which leads to partial silencing of the frataxin gene. FRDA patients and frataxin animal knock-outs have Fe^3+^ accumulation and oxidative stress which become more severe with aging^15–17^. It has long been debated whether oxidative stress is the cause of FRDA or the consequence after a long series of primary events^17^. The FRDA disease is thus an optimal case study which could strongly benefit from the nanosensor technology to explore in detail the early stages of disease and follow the metabolic status of FRDA cells. Using the CRISPR/Cas9 technology, we developed in the past a mammalian cell line (HEK-*cFXN*) which can produce different frataxin levels by switching off partially or completely the *FXN* gene^18^. This system, which was the first after the exploratory study by Ristow and coworkers to use mammalian cells^19^, allowed us to introduce a temporal dimension to the study the early events of FRDA. In a previous study that relied on traditional techniques such as fluorescence analysis and enzymatic assays, we showed that overexpression of the frataxin gene affects the cellular metabolism and leads to a significant increase of oxidative stress and labile iron pool levels. These cellular effects are similar to those observed when the gene is partially silenced, as it is in FRDA patients^20^.

We demonstrated that the nanosensor is a powerful and flexible technique able to provide important information on the metabolic changes caused by frataxin deficiency which is in agreement with and complement the conclusions obtained by conventional techniques. We showed that the cell has a clear response to frataxin overexpression under oxidative stress conditions that is characterized by multiple steps of metabolic activity. We validated our conclusions with fluorescence measurements that provided a complementary but coherent perspective. The information obtained by the nanosensor, coupled with more traditional techniques, can thus help us to study the metabolic landscape of the cellular response. We envisage that this technique will turn in the future into an important tool able to assist FRDA studies and eventually be exploited to inform on the effects of new therapeutics directly on patients’ cells.

## Materials and Methods

### Cellular model

The cellular model, HEK-*cFXN*, used in this study is a derivative HEK293 cell line characterised by a CRISPR-induced biallelic knockout of the endogenous *FXN* gene and the presence of an exogenous, inducible cDNA *FXN* cassette (*cFXN*). The *cFXN* gene is under the control of a tetracycline regulated promoter (CMV-TetO2) that allows switching off/on the *cFXN* gene^18^. This system can result in induction of increasing levels of the protein as a function of the tetracycline concentration. The HEK-*cFXN* model has been previously used to study the effects of frataxin overexpression: cells cultured in the presence of 10 or 100 ng/ml tetracycline have levels of frataxin expression substantially increased respect to endogenous frataxin levels of wildtype HEK293 cells (13- and 17-fold increase, respectively). In both cases, the frataxin levels progressively decrease upon removal of tetracycline from the culture medium, reaching physiological levels after an 8-day period^20^. In the present study, we used HEK-cFXN cells grown in 10 (10-tet-HEK-*cFXN*) or 100 (100-tet-HEK-*cFXN*) ng/ml tetracycline as models for mild (10 ng/ml) and strong (100 ng/ml) frataxin overexpression. Cells at the 8-day period (8-day-HEK-*cFXN*) were considered as internal control cells for each of the overexpression levels since they produce basal frataxin levels of HEK cells. For each independent experiment the basal level was assumed the average response observed in the period before inducing an oxidative stress response.

### Cell culture

Cell culture and reagents were acquired from ThermoFisher Scientific (Waltham, MA, USA). HEK-cFXN cells were cultured in Dulbecco modified Eagle medium, supplemented with 10% tetracycline-free fetal bovine serum (FBS; Clontech, Takara), 10 mM sodium pyruvate, 2 mM L-glutamine, 2% (v/v) non-essential amino acids (NEAAs), 100 μg/ml hygromycin B and 15 μg/ml blasticidin. Cells were kept in an incubator (Binder CB 150) at 37 °C in humidified air enriched with 5% CO_2_.

During the experiments, the culture medium was supplemented with either 10 or 100 ng/ml of tetracycline (Sigma-Aldrich, St. Louis, Missouri, USA) to obtain respectively mild and strong frataxin overexpression. Oxidative stress conditions were triggered by initially culturing cells overnight in the presence of 50 μM ferric ammonium citrate (mild iron overload) followed by 30 min of 300 µM H_2_O_2_.

### Nanomotion assays

We employed a Nanosurf FlexAFM 5 microscope to monitor the metabolically-related cellular fluctuations. The microscope was mounted on an Olympus IX50 inverted optical microscope (Olympus Inc., Tokyo, Japan) and connected to a National Instruments card for direct collection of the nanomotion signal. The AFM device was equipped with commercial cantilevers (Bruker DNP-10, nominal spring constant 0.12 N/m) modified through chemical functionalization to ensure cell attachment to the sensor: the tips were exposed for 15 minutes to poly-D-lysine (20 µg/ml, Sigma-Aldrich), followed by rinsing, air drying and immediate use. This functionalization protocol has been demonstrated most suitable for cellular attachment^21,22^. The AFM device was inserted in the incubator to ensure that the cells under investigation would be kept under controlled temperature and CO_2_ conditions. We then used the AFM motors to move the functionalized sensor in the near proximity of a cell and applied on it a small pressure (5 nN). This procedure ensured attachment of the cell on the sensor, which was then retracted and used for the nanomotion experiment. We exploited conventional optical microscopy to determine the number and position of the attached cells on the cantilever sensor. In a typical nanomotion experiment, we collected continuously the oscillations of the sensor produced by the metabolic activity of the attached cells in several independent 30-minute periods over approximately 1–2 hours with an acquisition rate of 20 kHz. We first measured the cells in the presence of standard culture medium. We then added the oxidising agent (at a final concentration of 300 µM H_2_O_2_) to evaluate cell response over time, over more than 40 minutes. We coupled the nanosensor measurements with optical images collected by the inverted microscope using a 40x objective.

### Data analysis

The recorded fluctuations appeared as coloured noise patterns. We analysed the data by calculating the variance of the vibrations over time using a custom LabView software that performed the analysis in several steps. At first, the data were split in 30 second sets and a simple linear fit was applied to each set. Next, the variance within each set was calculated and separated according to whether the data were collected before and after the addition of the oxidising agent. We averaged the data points before the addition of the H_2_O_2_ and tagged the resulting value as 100% to evaluate the effect of the stimulus and allow comparison between the different experiments. The data after exposure to the oxidising agent were then averaged in groups representing data collected within 3–5 minutes to reconstruct the variance variation vs. time. All experiments were performed at least in triplicates using independently prepared cells and over different time-periods. The graphs presented are the average of minimally three experiments performed under the same conditions.

### MitoSOX red assays

Cell samples were incubated for 20 min at 37 °C with MitoSOX Red (Thermo Fisher Scientific, Waltham, MA, USA) at a concentration of 1.25 μM. Measurements were carried out on 10^4^ cells per sample by flow cytometry (BD Fortessa, excitation/emission at 488 nm and 710 nm, respectively) and the results are expressed as percentages of fluorescent cells on the whole population. Results are expressed as means of three to five independent samples for each condition. Statistical significance was tested using one-way ANOVA and Dunnett post hoc test (**P ≤ 0.0045; ****P ≤ 0.0001).

## Results

### Measurement of the cellular motion at the nanoscale

**Figure 1:**
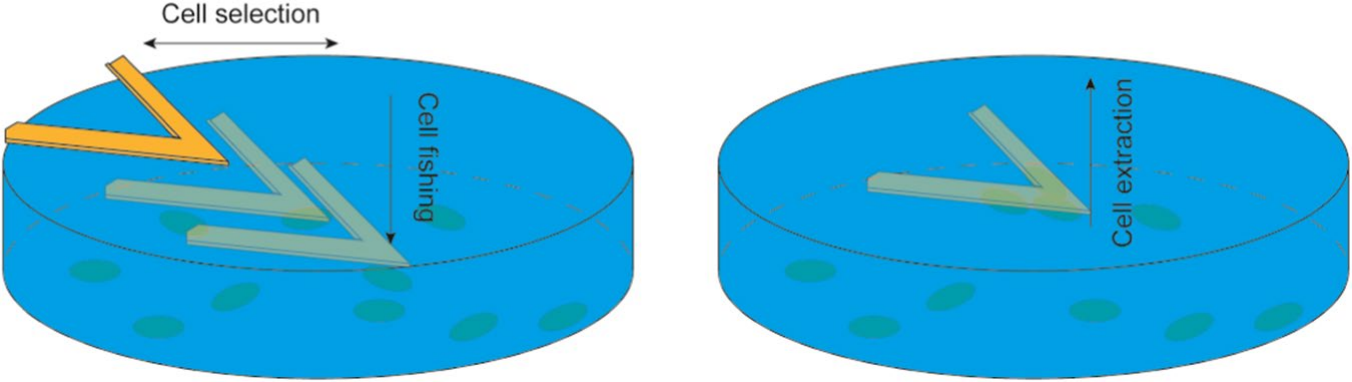
Depiction of the immobilization protocol of the HEK cells on the nanosensor. The sensor is monitored by optical microscopy to approach a cell. The chemically-activated surface of the sensor is pressed over the cell for 1 minute in order to ensure attachment and then retracted to extract the cell. This procedure is repeated in order to obtain 3–5 cells over a single sensor to perform the nanomotion experiment.

The nanosensor consists of a cantilever on which cells are attached, following protocols developed previously^9^ (Fig. 1). The data are recorded as the amount of cantilever fluctuations as a function of time. We proved in the past that these movements correlate directly with the increasing and decreasing of the metabolic activity of the cells attached to the sensor’s surface^4,5^. Deviations from the baselines indicate cell viability and their intensities will indicate how much the cells treated by specific environmental insults differ from the controls in terms of metabolic behaviour.

**Figure 2:**
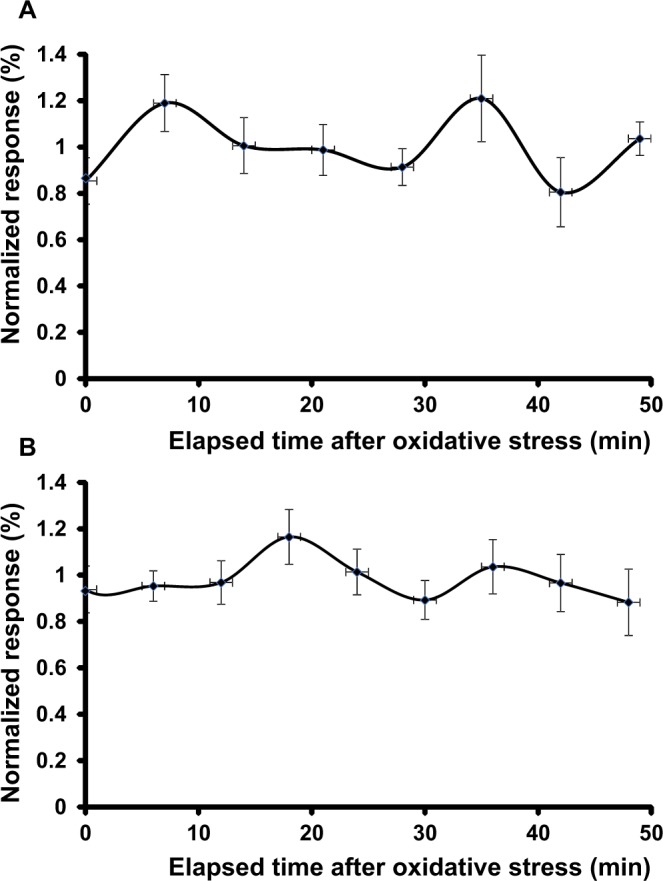
Control experiments for the establishment of the method. Measurements on (**A**) 10-tet-HEK-*cFXN* and (**B**) 100-tet-HEK-*cFXN* cells in a medium containing ferric ammonium citrate recorded over 50 minutes time. The response was normalized to the average value calculated on the fluctuations in culture medium without addition of peroxide.

We first performed control experiments to establish the feasibility of the technique and define the default oscillations of the cellular response to the geometry of the setup and the functionalization of the sensor. We incubated 10-tet-HEK-*cFXN* and 100-tet-HEK-*cFXN* cells on the cantilever in the analysis chamber at 37 °C. These assays showed how the cells survived on the sensor for more than one hour with little variations, both in morphology and nanomotion response (Fig. 2).

### The effects of oxidative stress on HEK-*cFXN* cells

**Figure 3:**
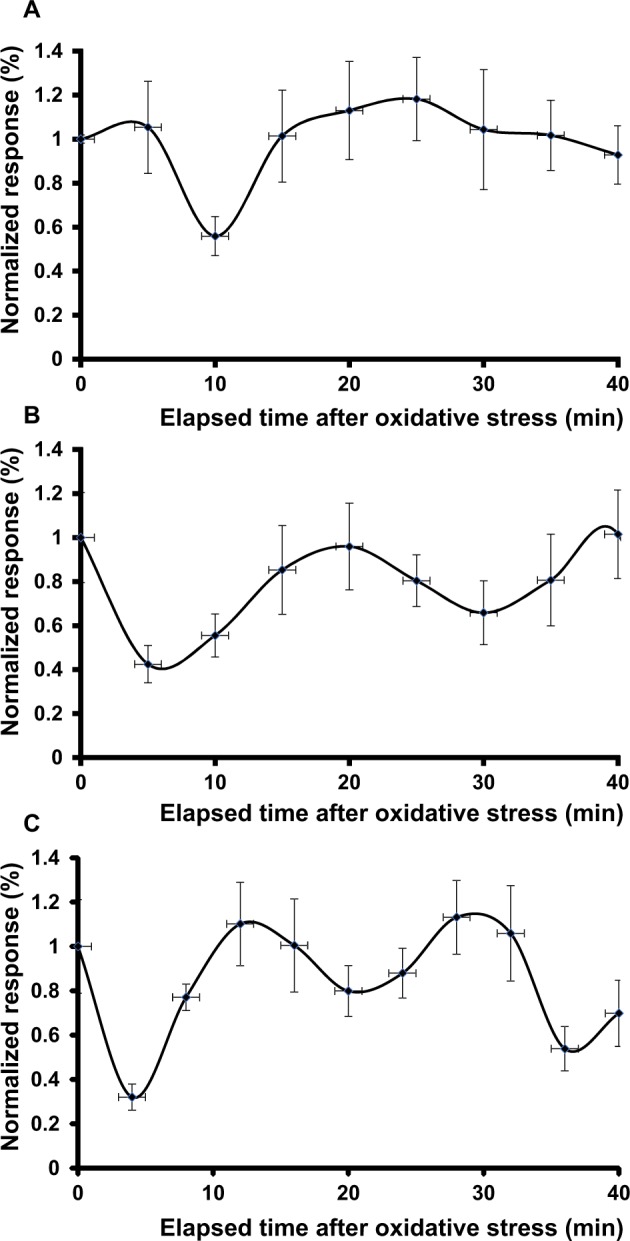
Effects of hydrogen peroxide on HEK-*cFXN* cells. Nanomotion experiments of the (**A**) 8-day-HEK-*cFXN*; (**B**) 10-tet-HEK-*cFXN*; (**C**) 100-tet-HEK-*cFXN* cells exposed to oxidative stress. The cells were cultured overnight in the presence of ferric ammonium citrate (50 μM) and then exposed to H_2_O_2_ to a final concentration of 300 μM after a stabilizing period of 1 h in growth medium.

Next, we monitored the cells when exposed to oxidative stress conditions. To evaluate the role of ferric ammonium citrate that mimics the condition of iron overload observed in FRDA patients, we performed nanomotion response experiments by comparing cells incubated in the presence of this sensitizing agent adding peroxide. The cells were all cultured overnight in the presence of ferric ammonium citrate (50 μM). A cell sample was then collected by the cantilever and the measurement started. After a first stabilizing period of 1 h in growth medium, which set the average level of fluctuations for the specific condition, we introduced in the analysis chamber H_2_O_2_ to achieve a final concentration of 300 μM. Depending on the levels of frataxin expression, the cells had strikingly different responses (Fig. 3). Ten minutes after exposure to oxidative stress, the control cells 8-day-HEK-*cFXN* showed a sharp reduction of the nanoscale movements (0.4%) which lasted less than 10 minutes and rapidly returned to the basal levels (i.e. the levels observed after a stabilizing period of 1 h) (Fig. 3A). This is indicative that HEK-*cFXN* cells with endogenous frataxin levels are able to recover from oxidative stress rapidly and maintain a normal metabolic activity also in the presence of oxidative agents.

The cells with frataxin mild overexpression (10-tet-HEK-*cFXN* cells) had a more complex response (Fig. 3B). The response to H_2_O_2_ in this case was faster and more accentuated since the movements more rapidly (after 5 minutes) dropped by 0.6 units the signal observed before the peroxide insult. The response took almost 20 minutes to recover partially before entering in a second shallower minimum (a 0.35 units drop). Finally, the cells recovered rapidly (after less than 10 minutes), stabilizing the signal to values comparable to the basal level.

The cells with strong frataxin overexpression (100-tet-HEK-*cFXN* cells) also showed a nanomotion pattern with two minima. The data showed a sharp decrease of cellular movement with a minimum (a 0.7 units drop ca. 3 minutes after peroxide addition), which lasted 10 minutes (Fig. 3C**)**. This behaviour was followed by a second shallower and wider minimum (0.2 units drop at 14 minutes after the insult) and third one almost as deep as the first (0.50 units drop) 36 minutes after peroxide addition. In this case the cell response did not recover after 40 minutes indicating a more complex response pattern of these cells to oxidative agents, which was not completed at the end of the recording time.

**Figure 4:**
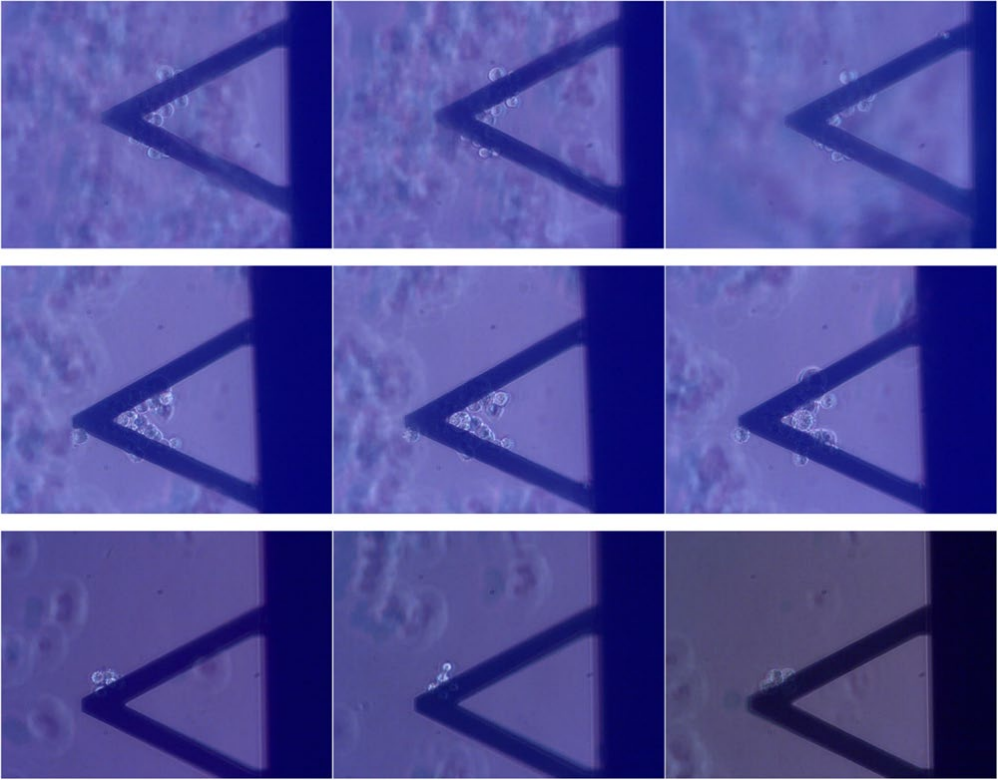
Effects of hydrogen peroxide on the cell morphology. Optical images of the 8-day-HEK-*cFXN* (top panels), 10-tet-HEK-*cFXN* (middle panels) and 100-tet-HEK-*cFXN* (lower panels), before (left), 5 minutes (center) and 40 minutes (right) after exposure to peroxide. The images show that there is no evident morphological modifications of the cells.

Notably, all response patterns were not associated to morphological indications of cell suffering or cell death as shown by optical images (Fig. 4 for 8-day-HEK-*cFXN*, 10-tet-HEK-*cFXN* and 100-tet-HEK-*cFXN* respectively).

### Effect of peroxide in the absence of iron overload

**Figure 5:**
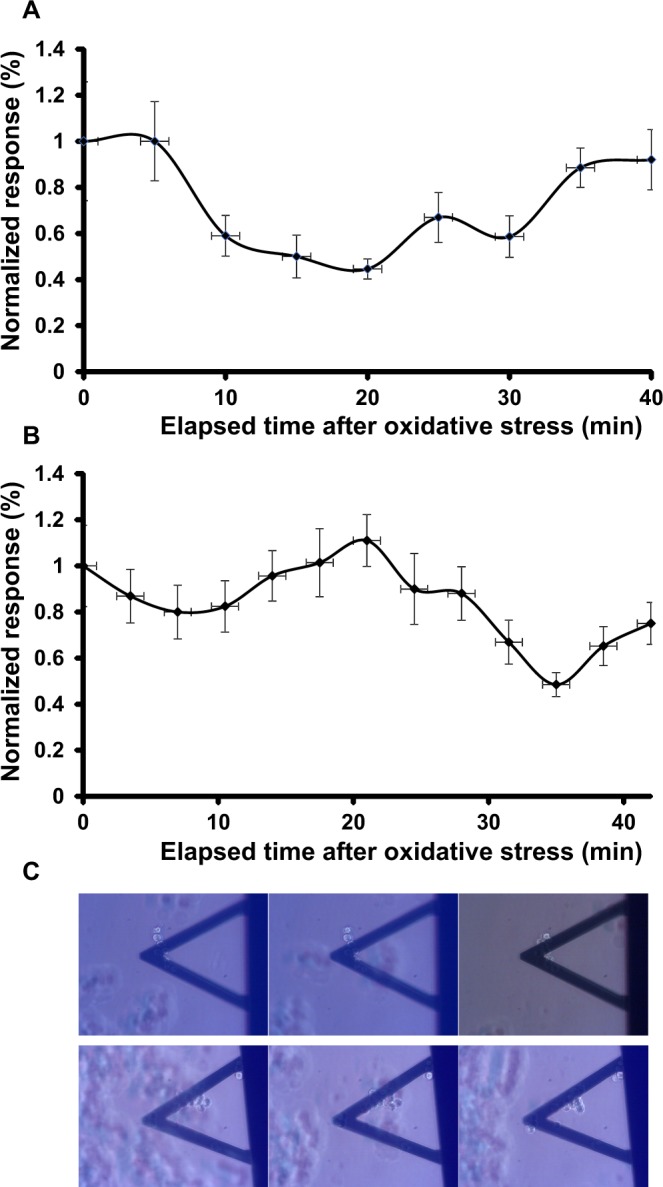
Effects of hydrogen peroxide in the absence of ferric ammonium citrate. Nanomotion experiments of (**A**) 10-tet-HEK-*cFXN* and (**B**) 100-tet-HEK-*cFXN* cells exposed to oxidative stress. The response was normalized to the average value calculated on the fluctuations of the corresponding medium. (**C**) Optical images of 10-tet-HEK-*cFXN* (top panels) and 100-tet-HEK-*cFXN* (lower panels), before (left), 5 minutes (center) and 40 minutes (right) after exposure to H_2_O_2_.

We then controlled the effects of the peroxide without preincubation in ferric ammonium citrate. The 10-tet-HEK-*cFXN* cells had a response similar to that of the 10-tet-HEK-*cFXN* cells incubated with ferric ammonium citrate evidencing two minima (Fig. 5A). As for 8-day-HEK-*cFXN*, 5 minutes were needed to see a response to H_2_O_2_, but in this case the reduction of the movement was less intense and lasted longer (more than 20 minutes). Following the first minimum, the cell response had a second minimum of shorter length (15 minutes approximately). It is worth highlighting how the two minima were shallower, suggesting that the cell response was not as strong as for the exposure to ferric ammonium citrate but more diffuse.

The 100-tet-HEK-*cFXN* cells also showed a two-minimum nanomotion pattern (Fig. 5B). The first minimum was less intense (a 0.2 units drop after ca. 8 minutes) as compared to those measured in the presence of ferric ammonium citrate and lasted ca. 15 minutes. As for the 10-tet-HEK-*cFXN* cells, the response was slower and required almost ten minutes to reach a minimum. The second minimum was deeper (a 0.5 units drop after ca. 35 minutes) than the first.

Overall, these experiments demonstrated that cells which were not sensitized with ferric ammonium citrate appeared to respond less intensely and slower to oxidative stress. Also in these experiments, we did not observe large-scale morphological changes indicating cell suffering or cell death (Fig. 5C).

### Detection of oxidative stress by fluorescence

**Figure 6:**
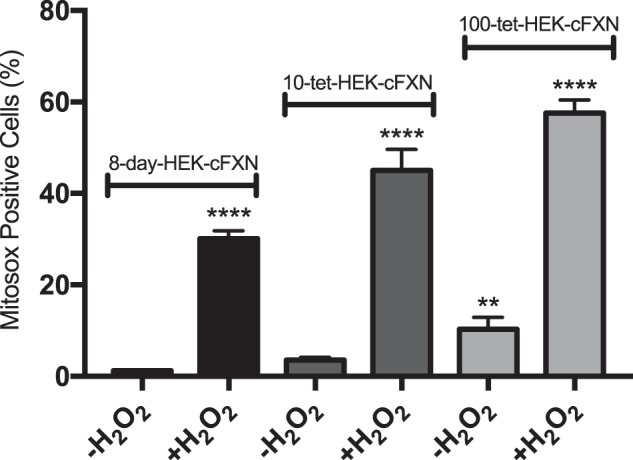
Measurements of oxidative stress by flow cytometry. The experiments were carried out using 1.25 μM MitoSOX Red on both untreated cells (-H_2_O_2_) and on cells under acute oxidative condition (+H_2_O_2_). Frataxin overexpression has a clear negative effect on the response of the cells to oxidative stress conditions. This effect, although at a lower level, can be seen even when cells are not treated with H_2_O_2_. Data expressed as means ± SD (n = 3/5). **P ≤ 0.0045 and ****P ≤ 0.0001 compared to 8-day control sample.

We have previously described the effects of oxidative stress on HEK-*cFXN* cells^20^. Overall, our previous results are in excellent agreement with the nanosensor experiments. To further validate the nanomotion technology, we adopted the widely used MitoSOX Red, a fluorescent dye used for the detection of ROS formation specifically in mitochondria^23^. Detection of ROS formation via MitoSOX Red fluorescence was performed not only on samples exposed to ferric ammonium citrate (50 μM) and H_2_O_2_ (300 μM) but also on untreated cells (Fig. 6). As expected, the increase in ROS production was particularly noticeable in samples exposed to oxidative conditions, with the strong overexpression sample (100-tet-HEK-*cFXN*) showing the highest response. The mild overexpression sample (10-tet-HEK-*cFXN*) was also clearly affected by the presence of the oxidising agent although at a lower intensity than the 100-tet-HEK-cFXN sample. An increase in MitoSOX Red fluorescence, albeit at a decisively lower intensity, was also detected in untreated 100-tet-HEK-*cFXN*. No significant difference from the control was evident for the mild overexpression samples (10-tet-HEK-*cFXN*).

## Discussion

We have shown here how the nanomotion sensor can be adapted and optimised to follow the metabolic landscape of cells designed to mimic a specific disease or explore the effects of genetic modifications. FRDA, a genetic disease linked to iron accumulation and metabolic variations, was chosen as a particularly suitable case study in view of the several debates on the role of oxidative stress as a primary cause or a secondary effect of the FRDA pathology^1,17^. We had previously studied the effects of frataxin overexpression because this is a possible strategy that could be used to cure the disease. Lack of frataxin is lethal, as shown by early studies that have demonstrated that mice knockout models die at the embryonal level^24^. Accordingly, the disease starts appearing when the frataxin levels are below 30% those of healthy controls and are 10–35% the normal levels in FRDA patients^15^. On the other hand, it was found that frataxin overexpression is not a solution as a therapeutic strategy: overexpression seems to be as toxic as its partial depletion^20,25,26^. This observation is perfectly coherent with the current hypothesis that frataxin functions as a regulator of the process of conversion of cysteine into alanine through interaction with the desulfurase central to the machine of the iron sulfur cluster biogenesis machine^27^.

Our interest here was to explore if the nanosensor could give us more details on the basis of the observed toxicity of overexpression and precise hints on when the effects start to be observable.

We first optimised the technique for our specific type of cells. The setup we implemented is inexpensive, small and easy to handle. It will thus be amenable to a wider and more standardised use in the future. Some aspects may be improved: in our current setup, the time span covered in the experiments is limited by the evaporation of the medium in the measuring chamber. Currently the plate contains small volumes (1 ml) which are needed to optimise the diffusion rate of peroxide. The problem can, in the future, be reduced or solved by the use of a perfusion chamber similar to those that have been employed in previous studies^28^.

We observed that frataxin overexpression has profound metabolic effects that are not linear. Our data clearly showed that increased levels of frataxin overexpression negatively affect the ability of the cell to respond to oxidative stress, also in the absence of iron overload. All cells evidenced a response to H_2_O_2_ as it could be expected. However, the 8-day-HEK-*cFXN* control recovered rapidly and had relatively small perturbations, while the other cells showed a more complex and stronger response. Both mild and strong frataxin-overexpression samples displayed a series of minima in response to the stress in general more intense than those of the control. The 100-tet-HEK-*cFXN* cells treated both with ferric ammonium citrate and peroxide were the only cases in which, at the end of the monitoring period, the basal movement did not recover and was still responding with a third minimum. This observation suggested that these cells need more time to recover and have a stronger sensibility to oxidative stress. It is interesting to highlight how the oscillation amplitude of the nanomotion signal of the 100-tet-HEK-*cFXN* cells also increased above basal levels..

In the whole, the nanomotion results are fully consistent with what we could observe by more conventional techniques as proven here by MitoSOX experiments and as previously shown by other techniques^20^. These more traditional approaches are nevertheless unable to monitor when the complex metabolic response of the cell take place. Conversely, the nanosensor measurements do not tell us which metabolic reactions occur but provide a direct profile of when the changes occur and add a time-resolved perspective on the cellular response to environmental insults. The nanosensor results tell us, for instance, that the effects of ROS on the metabolism protract for longer than the 30 min windows explored in the MitoSOX red measurements, an information that could not be extracted otherwise. The nanosensor measurements thus provide direct information on the metabolic landscape of the cellular response. In the future, it will be appropriate to couple nanomotion experiments with traditional approaches such as fluorescent markers to obtain first a more complete picture of what happens to the cell in real time and then explore with other means what is happening at the time points indicated by the nanosensor. This new possibility will open a new way to test the effects of drugs on cell models of FRDA and evaluate the effects produced by induced oxidative stress on the cellular metabolic activity. It is not farfetched to predict that the nanomotion sensor may be applied more in general to the study of neurodegenerative diseases, such as Parkinson and Alzheimer diseases, and be in a not too far future exploited as a powerful tool to explore the response to drugs of patients’ cell cultures in personalized medicine.
